# The predictive value of Tp−Te interval, Tp−Te/QT ratio, and QRS‐T angle of idiopathic ventricular tachycardia in patients with ventricular premature beats

**DOI:** 10.1002/clc.23998

**Published:** 2023-02-21

**Authors:** Wei Zhu, Xingmei Huang, Lili Mei, Liting Zhi, Shili Jiang, Jianling Jin, Cao Zou

**Affiliations:** ^1^ Department of Cardiology The First Affiliated Hospital of Soochow University Suzhou China

**Keywords:** idiopathic ventricular premature beats, idiopathic ventricular tachycardia, Tp−Te interval, Tp−Te/QT ratio, QRS‐T angle

## Abstract

**Background:**

Identify idiopathic ventricular tachycardia in patients with ventricular premature beats was required to have effectively treatment.

**Hypothesis:**

The aim of this study is to investigate the predictive value of Tp−Te interval, Tp−Te/QT ratio, and QRS‐T angle of idiopathic ventricular tachycardia in patients with idiopathic ventricular premature beats.

**Methods:**

One hundred and seventy‐eight patients who had undergone premature ventricular complex/ventricular tachycardia (PVC/VT) ablation between January 1, 2020 and August 30, 2022 constituted our study population as ventricular arrhythmia group. Seventy‐five healthy people were selected as control group. Patients with no episode of VT were classified as PVC group, while with any episode of VT that has the same morphology with PVC were classified as PVC with VT group. Patients in PVC with VT group were divided into two groups: nonsustained VT group (duration of any episode of VT below 30 s) and sustained VT group (duration of any episode of VT over 30 s). Tp–Te interval, Tp–Te/QT ratio and QRS‐T angle were compared in groups.

**Results:**

Tp–Te interval, Tp–Te/QT ratio and patients with increased QRS‐T angle in PVC with VT group were higher or more than those in PVC group (*p* < .001). The value of combined diagnosis of these indexes was higher. Tp–Te interval was longer in the sustained VT group compared to the nonsustained VT group (*p* = .009).

**Conclusion:**

Tp–Te interval, Tp–Te/QT ratio, and QRS‐T angle may have a predictive value of presence of idiopathic VT in patients with premature beats and the combined prediction of these indexes is more valuable. Tp–Te interval maybe helpful for prediction of sustained idiopathic VT.

## Graphical abstract

Tp‐Te interval, Tp–Te/QT ratio, and QRS‐T angle may have a predictive value of presence of idiopathic ventricular tachycardia (VT), and the combined prediction of these indexes is more valuable. Tp–Te interval maybe helpful for prediction of sustained idiopathic VT.

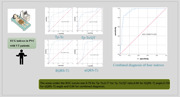

## 1 INTRODUCTION

Ventricular arrhythmia includes ventricular premature beat, ventricular tachycardia, ventricular flutter, and ventricular fibrillation. The common causes are structural heart disease and ion channel disease. It is also common in patients without structural heart disease.^1^

Idiopathic ventricular premature beat/ventricular tachycardia refers to ventricular arrhythmia without organic heart disease, electrolyte abnormality, and abnormal ion channel function. Most of them originate from left or right ventricular outflow tract.^2^, ^3^ Long‐term and high‐load idiopathic ventricular premature ventricular complex/ventricular tachycardia (PVC/VT) can also lead to tachycardia cardiomyopathy and affect cardiac function.^4^ Among them, sustained idiopathic VT can lead to syncope or sudden death in severe cases.

Tp−Te interval refers to the interval from peak of T wave to end of T wave. Tp−Te/QT ratio refers to the ratio of ventricular relative refractory period to total refractory period.^5^ QRS‐T angle reflects the relationship between ventricular depolarization and repolarization, which can be divided into frontal QRS‐T angle (f(QRS‐T)angle) and spatial QRS‐T angle (s(QRS‐T)angle).^6^ These indexes are closely related to ventricular repolarization dispersion. It is reported that these indexes are related to the occurrence of ventricular arrhythmia caused by organic heart diseases.^7^, ^8^ However, the relationship between these indexes and idiopathic ventricular arrhythmia is seldom reported. The aim of this study is to investigate the predictive value of Tp−Te interval, Tp−Te/QT ratio, and QRS‐T angle of idiopathic ventricular premature beats in patients with idiopathic ventricular premature beats.

## 2 MATERIAL AND METHODS

### 2.1 Ethical issues

The single‐center retrospective study was performed in full accordance with the principles outlined in the Declaration of Helsinki, and permission was obtained from the ethics committee of Soochow University.

### 2.2 Patients and study design

This study was a retrospective cohort analysis. A total of 178 patients (50.67 ± 17.9 years, 89 male) without any exclusion criteria who had undergone PVC/VT ablation between January 01, 2021 and August 30, 2022. All the selected patients did not use antiarrhythmic drugs or stopped using antiarrhythmic drugs for five half lives before ablation. Ventricular premature beat or ventricular tachycardia that has the same morphology with PVC were recorded by Holter. Exclusion criteria: (1) Patients with severe liver and kidney disease, electrolyte abnormality, thyroid dysfunction, nervous system disease, and diabetic patients with poor blood glucose control; (2) Patients with coronary heart disease, cardiomyopathy, congenital heart disease, valvular heart disease, and other structural heart diseases; (3) Patients with other tachyarrhythmia or bradyarrhythmia, such as atrial fibrillation, sick sinus syndrome, and so forth. At the same time, we selected 75 cases for physical examination in our hospital as the control group and matched with the gender, age, and other factors of the premature ventricular arrhythmia group.

### 2.3 Research methods

All the patients received 12 lead ECG (Mac,2000) and Holter (Boying, BI6812, BI9100). Patients with no episode of VT were classified as PVC group (114 patients) while with any episode of VT that has the same morphology with PVC were classified as PVC with VT group (64 patients) in the ventricular arrhythmia group. Patients in PVC with VT group were divided into two groups: nonsustained VT group (duration of any episode of VT below 30 s, 31 patients) and sustained VT group (duration of any episode of VT over 30 s, 33 patients). Tp–Te interval, Tp–Te/QT ratio, and QRS‐T angle were measured or calculated. The Tp–Te interval was measured in the V6 lead. The peak of T wave is the highest point of T wave and the terminal part of T wave is the intersection point between the baseline and the descending branch of T wave. If lead V6 is unstable or is not suitable for measurement, lead V4 or V5 can be selected. QT interval is the interval from the starting point of the QRS complex to the end point of the T wave. QTc interval is calculated by Bazetts formula. QRS‐T angle reflects the relationship between ventricular depolarization vector and repolarization vector, which can be divided into: (1) spatial (s[QRS‐T]): spatial QRS‐T angle is defined as the angle between QRS‐ and T‐wave vectors in three‐dimensional (3D) space. s(QRS‐T) angle is measured at the moment of maximum magnitude of the spatial QRS vector and T vectors within a 3D QRS loop and T loop. We get s(QRS‐T) angle by ECG system (Nalong, China). (2) Frontal QRS‐T angle ((f)QRS‐T): frontal QRS‐T angle is the projection of spatial QRS‐T angle onto the frontal plane and it was calculated as the absolute value of difference between QRS‐and T‐axis. f(QRS‐T) > 90° or s(QRS‐T) > 105° is abnormal.

### 2.4 Statistical methods

Spss 26.0 software was used for statistical analysis. Data were expressed as mean±standard deviation. Categorical variables were expressed in ratio. Differences between groups were tested by *t* tests for unpaired data once normality was demonstrated and the categorical variables were analyzed by *χ* ^2^ test. Receiver operating characteristic (ROC) curves were generated for evaluation of QTc, Tp−Te interval, Tp−Te/QT ratio, and QRS‐T angle in predicting the presence of idiopathic VT.

## 3 RESULTS

### 3.1 QTc, Tp−Te interval, Tp−Te/QT ratio, and QRS‐T angle in control group and patients with idiopathic ventricular arrhythmia

Clinical baseline of subjects are displayed on Supporting Information: Table 1. QTc, Tp−Te interval, and Tp−Te/QT ratio in idiopathic ventricular arrhythmia group were significantly higher than those in control group (440.37 ± 41.50 vs. 412.15 ± 41.5, 89.85 ± 16.13 vs. 80.29 ± 10.58 ms, 0.23 ± 0.43 vs. 0.21 ± 0.03) (*p* < .001). The people with increased f(QRS‐T) angle and s(QRS‐T) angle in the idiopathic ventricular arrhythmia group were more than that in the control group (44 cases, 24.7% vs. 6 cases, 8.0%, *p* = .002, 63 cases, 35.4% vs. 12 cases, 16.0%, *p* = .002) (Table 1).

**Table 1:** Comparison of QTc, Tp−Te, and Tp−Te/QT ratio between two groups.

	Control group	Isolated PVC/VT	T/x^2^ value	*p* Value
QTc (ms)	412.15 ± 41.50	440.37 ± 41.50	6.818	<.001
Tp−Te (ms)	80.29 ± 10.58	89.85 ± 16.13	5.560	<.001
Tp−Te/QT	0.21 ± 0.03	0.23 ± 0.43	4.563	<.001
f (QRS‐T) angle > 90°, *n*(%)	6 (8.0%)	44 (24.7%)	9.303	.002
s (QRS‐T) angle > 105° *n*(%)	12 (16.0%)	63 (35.4%)	9.515	.002

*Note*: Values reflect mean ± standard deviation or *n*(%). Significant differences in comparisons (*p* < .05).

Abbreviations: PVC, premature ventricular complex; VT, ventricular tachycardia.

### 3.2 QTc interval, Tp−Te interval, Tp−Te/QT ratio, and QRS‐T angle in PVC group and PVC with VT group

**Figure 1:**
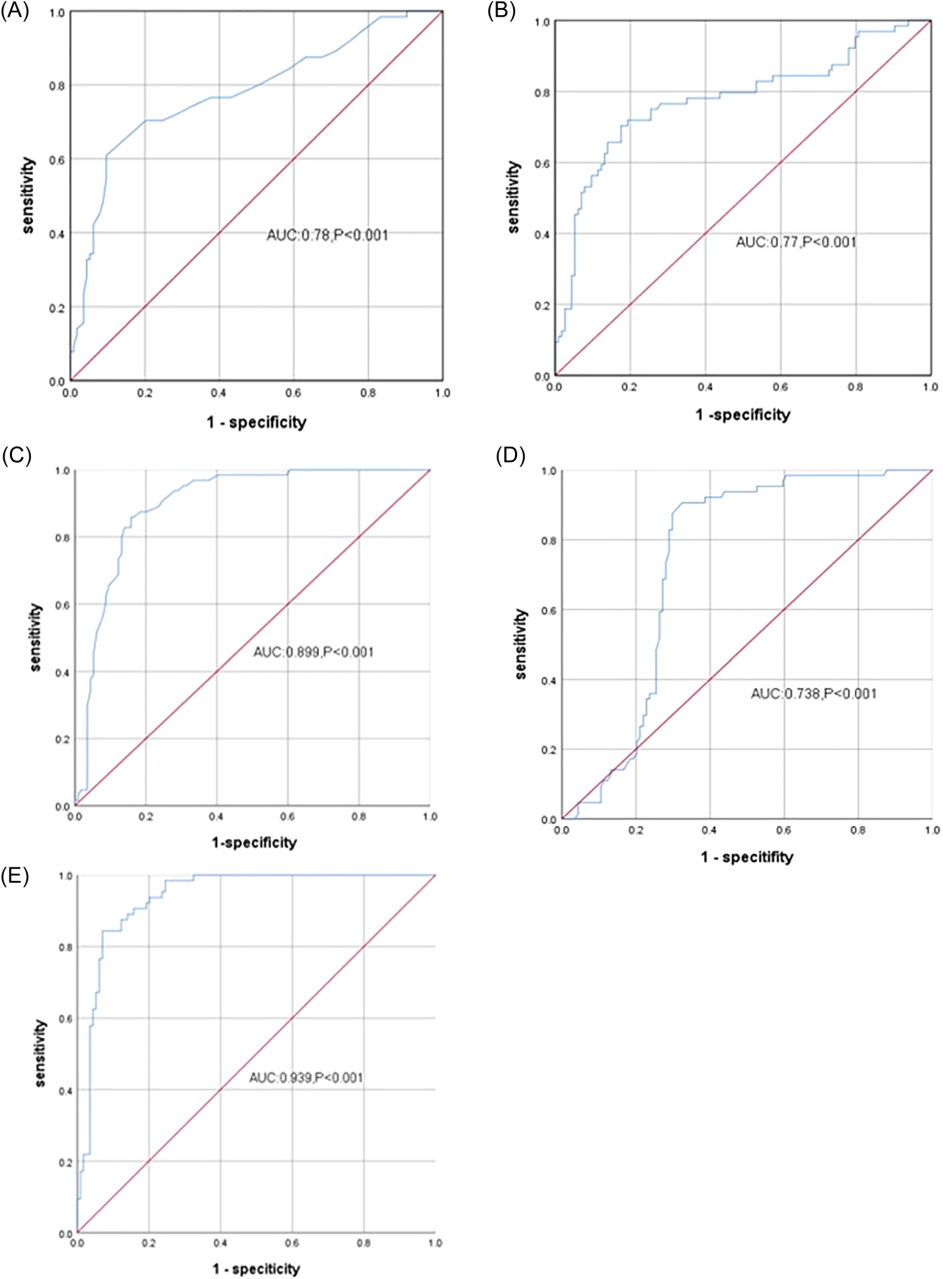
ROC curves. Tp−Te, Tp−Te/QT ratio, f(QRS‐T) angle, s(QRS‐T angle), and combined diagnosis with multiple indexes ROC curves. The areas under the ROC curves was 0.78 for Tp−Te, 0.77 for Tp−Te/QT ratio, 0.90 for f(QRS‐T) angle, 0.734 for s(QRS‐T) angle, and 0.94 for combined diagnosis. (A) ROC for Tp‐Te, (B) ROC for Tp‐Te/QT, (C) ROC for f(QRS‐T) angle, (D) ROC for s(QRS‐T) angle, (E) ROC for combined diagnosis of Tp‐Te, Tp‐Te/QT, f(QRS‐T) angle and s(QRS‐T) angle. ROC, receiver operating characteristic.

QTc had no significant difference in the two groups (434.97 ± 38.00 vs. 443.39 ± 31.44 ms, *p* = .179). Tp−Te interval, Tp‐Te/QT ratio in PVC with VT group were significantly higher than those in PVC group (99.79 ± 19.20 vs. 84.26 ± 10.69 ms, 0.25 ± 0.05 vs. 0.21 ± 0.03) (*p* < .001). The people with increased f(QRS‐T) angle and s(QRS‐T) angle in the PVC with VT group were more than that in the PVC group (30 cases, 46.9% vs. 14 cases, 12.3%, 33 cases, 51.6% vs. 29 cases, 25.4%) (*p* < .001) (Table 2). The areas under the ROC curves was 0.78 for Tp−Te, 0.77 for Tp−Te/QT ratio, 0.90 for f(QRS‐T) angle, 0.74 for s(QRS‐T) angle, and 0.94 for combined diagnosis (Figure 1).

**Table 2:** Comparison of QTc, Tp−Te interval, and Tp−Te/QT ratio between isolated PVC and isolated PVC with VT.

	Isolated PVC	VT	T/x^2^ value	*p* Value
QTc (ms)	443.39 ± 31.44	434.97 ± 38.00	1.303	.179
Tp−Te (ms)	84.26 ± 10.69	99.79 ± 19.20	5.974	<0.001
Tp−Te/QT	0.21 ± 0.03	0.25 ± 0.05	6.854	<0.001
f(QRS‐T angle) > 90° *n*(%)	14 (12.3%)	30 (46.9%)	26.361	<0.001
S(QRS‐T angle) > 135° *n*(%)	29 (25.4%)	33 (51.6%)	12.323	<0.001

*Note*: Values reflect mean ± standard deviation or *n*(%). Significant differences in comparisons (*p* < 0.05).

Abbreviations: PVC, premature ventricular complex; VT, ventricular tachycardia.

### 3.3 QTc interval, Tp−Te interval, Tp−Te/QT ratio, and QRS‐T angle in sustained and nonsustained VT group

**Figure 2:**
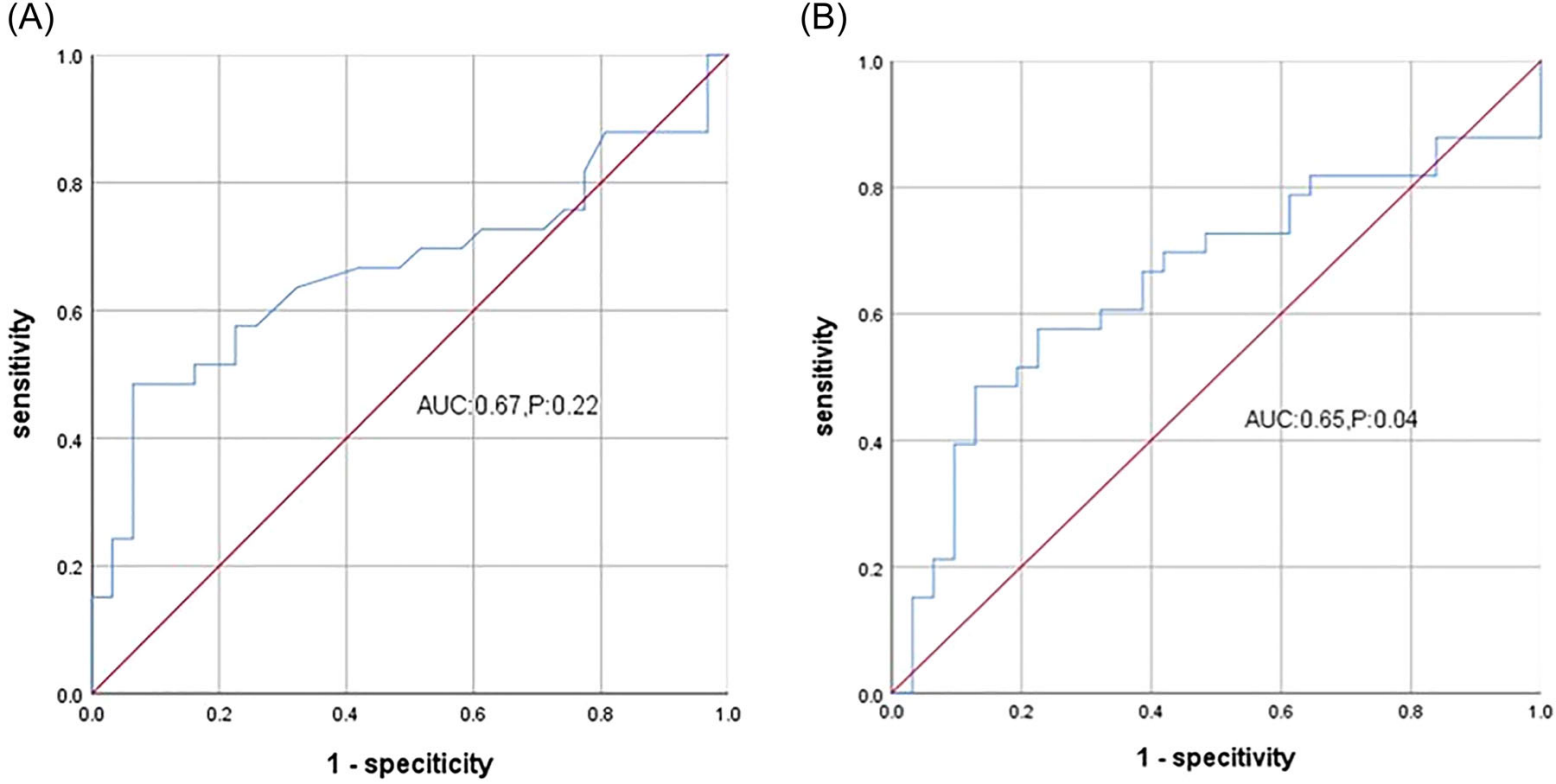
ROC curves. Tp−Te, Tp−Te/QT ratio ROC curves. The areas under the ROC curves was 0.67 for Tp−Te, 0.65 for Tp−Te/QT ratio. (A) ROC for Tp‐Te, (B) ROC for Tp‐Te/QT. ROC, receiver operating characteristic.

Tp−Te interval was significantly longer in sustained VT group than that in nonsustained VT group (105.70 ± 23.01 vs. 93.52 ± 11.25 ms, *p* = .009). There were no difference between other indexes (Table 3). The area under the ROC curves was 0.67 for Tp−Te (Figure 2).

**Table 3:** Comparison of QTc, Tp−Te, and Tp−Te/QT ratio between isolated PVC with sustained VT and nonsustained VT.

	None—sustained VT	Sustained VT	T/x^2^ value	*p* Value
QTc (ms)	434.26 ± 36.09	435.63 ± 40.26	0.144	.886
Tp−Te (ms)	93.52 ± 11.25	105.70 ± 23.01	2.708	.009
Tp−Te/QT	0.24 ± 0.04	0.26 ± 0.05	1.874	.066
f(QRS‐T) angle > 90° *n*(%)	13 (41.9%)	17 (51.5%)	0.589	.443
s(QRS‐T) angle > 135° *n*(%)	13 (41.9%)	20 (60.6%)	2.231	.135

*Note*: Values reflect mean ± standard deviation or *n*(%). Significant differences in comparisons (*p* < 0.05).

Abbreviations: PVC, premature ventricular complex; VT, ventricular tachycardia.

## 4 DISCUSSION

Tp−Te interval, Tp−Te/QT ratio, and QRS‐T angle are reported to be related to the occurrence of ventricular arrhythmia caused by organic heart disease, which are due to ventricular repolarization dispersion. Idiopathic ventricular arrhythmia usually showed a monomorphic pattern of beat or tachycardia, which is caused by triggered activity, mediated by delayed after depolarization. Most of idiopathic PVCs and VTs are originate from right or left outflow tracts. Triggered activity due to delayed after depolarization is suggested to be related with it. Overload diastolic intracellular Ca during Phase IV of the action potential maybe the reason.^9^

QT interval means the ventricular repolarization but is affected by heart rate. QTc interval is corrected QT interval by heart rate, but it still cannot reflect the real transmural dispersion of ventricular repolarization. Our research showed that QTc interval in the idiopathic ventricular arrhythmia group was longer than that in the control group, but there was no difference between PVC group and PVC with VT group, and between sustained and nonsustained VT group.

Tp−Te interval is the interval from the peak of T wave to the end of T wave. Tp represents the complete repolarization of epicardium, while Te represents the complete repolarization of M cells in the middle layer. Antzelevitch et al. found that Tp−Te interval is from the completed repolarization of epicardium of the left ventricular wall to the repolarization of M cells, which is related to the transmural repolarization dispersion of the whole ventricle.^10^ Increased Tp−Te interval is due to the high risk of ventricular arrhythmia. However, the body weight can affect the QT interval and Tp−Te interval. Tp−Te/QT ratio is a more stable index, which can eliminate the influence caused by mixed factors such as individual differences and heart rate. Ayhan Kup et al. reported that Tp−Te interval and Tp−Te/QT have certain value in predicting outflow tract ventricular tachycardia which is in accordance with our study.^11^ They also found that Tp−Te interval and Tp−Te/QT ratio decreased significantly after idiopathic outflow tract PVCs ablation.^12^ Our research showed that the Tp−Te interval and Tp−Te/QT ratio in the idiopathic ventricular arrhythmia group are higher than those in the control group. In the idiopathic ventricular arrhythmia group, the Tp−Te interval and Tp−Te/QT ratio in the PVC with VT group are higher than in the PVC group. The area under the ROC curve is more than 0.70, which shows that Tp−Te interval and Tp−Te/QT ratio may has a predictive value of presence of idiopathic VT. In the sustained VT group, Tp−Te is longer than in the nonsustained group, but the area under the ROC curve is 0.67. Tp−Te/QT ratio has no significant difference in sustained and nonsustained groups.

f(QRS‐T) angle over 90° and s(QRS‐T) angle over 105° is considered as a independent predictor of ventricular arrhythmia in patients with heart disease.^13^ The prognostic significance of increased QRS‐T angle is most likely to be due to an abnormal axis of the T‐wave, which reflects an abnormal sequence of ventricular repolarization which may lead to a fatal arrhythmia.^7^ But there are few reports about QRS‐T angle and idiopathic VT. Our study showed that patients with increased QRS‐T angle in idiopathic ventricular arrhythmia group were more than in control group, and were more in PVC with VT group than in PVC group, but there was no significant difference between sustained and nonsustained VT group.

In clinical practice, we found that some patients with ventricular premature beats will not affect the cardiac structure and function in a short time. However, some patients with idiopathic ventricular tachycardia have syncope, hypotension and even sudden cardiac death when tachycardia attacks. A few of them have ventricular tachycardia as their initial and only manifestation. Implantable cardioverter defibrillator or radio frequency ablation should be carried out for these patients. The need of careful follow‐up and using noninvasive tool to identify high‐risk patients was required to have effectively treatment.

## 5 CONCLUSION

Tp−Te interval, Tp−Te/QT ratio, and QRS‐T angle may has a predictive value of presence of idiopathic VT and the combined prediction of these indexes is more valuable. Tp−Te interval maybe helpful for prediction of sustained idiopathic VT.

## AUTHOR CONTRIBUTIONS

All authors contributed significantly to this study. Cao Zou and Wei Zhu designed the study. Xingmei Huang and Lili Mei collected the data. Liting Zhi and Shili Jiang analyzed the data. Jianling Jin made the Tables and Figures. Cao Zou and Wei Zhu wrote the paper. All authors reviewed and approved the manuscript.

## CONFLICT OF INTEREST STATEMENT

The authors declare no conflict of interest.

## Supporting information

Supplementary information.

## Data Availability

The datasets used and/or analyzed during the current study are available from the corresponding author on reasonable request.
