# Supplementary material for: The predictive value of Tp−Te interval, Tp−Te/QT ratio, and QRS‐T angle of idiopathic ventricular tachycardia in patients with ventricular premature beats
Source: Clin Cardiol. 2023 Feb 21;46(4):425–30. doi: 10.1002/clc.23998 (PMC10106654; doi:10.1002/clc.23998)
Supplement: Supplementary file 1 — Supplementary information. [file CLC-46-425-s001.docx]

Supplementary Table 1. Baseline clinical data of subjects

|  | Control group | Isolated PVC/VT | T/x^2^ value | P value |
| --- | --- | --- | --- | --- |
| Age(y) | 47.32±9.28 | 50.67±17.90 | 1.496 | 0.137 |
| Gender(male) n(%) | 44(58.7%) | 89(50.0%) | 1.696 | 0.193 |
| Hypertension n(%) | 12(16.0%) | 48(27.0%) | 3.508 | 0.061 |
| Diabetes n(%) | 7(9.2%) | 14(7.8%) | 0.136 | 0.712 |
| ACE inhibitor and Angiotensin receptor blocker n(%) | 12(16.0%) | 30(16.9%) | 0.028 | 0.868 |
| Beta blocker n(%) | 6(8.0%) | 16(9.0%) | 0.065 | 0.799 |
| Ca channel blocker n(%) | 9(12.0%) | 14(7.9%) | 1.092 | 0.296 |

Note: Values reflect mean±standard deviation or n(%).Significant differences in pairwise comparisons (p <0.05).

Abbreviations:PVC: Premature ventricular complex; VT: Ventricular tachycardia
